# Talking about breast symmetry in the breast cancer clinic: What can we learn from an examination of clinical interaction?

**DOI:** 10.1111/hex.13144

**Published:** 2021-01-31

**Authors:** Stephanie Mace, Sarah Collins, Susan Speer

**Affiliations:** ^1^ Division of Psychology and Mental Health School of Health Sciences The University of Manchester Manchester UK; ^2^ Division of Medical Education School of Medical Sciences The University of Manchester Manchester UK

**Keywords:** breast asymmetry, breast cancer, conversation analysis, medical interaction, psychosexual health

## Abstract

**Background:**

Breast asymmetry is a common post‐operative outcome for women with breast cancer. Quality of cosmetic result is viewed clinically as a critical endpoint of surgery. However, research suggests that aesthetic standards governing breast reconstruction can be unrealistic and may problematically enforce feminine appearance norms. The aim of reconstructive procedures is to help women live well with and beyond breast cancer. Therefore, understanding how patients and clinicians talk about surgical outcomes is important. However, we lack evidence about such discussions.

**Objective:**

To examine clinical communication about breast symmetry in real‐time consultations in a breast cancer clinic.

**Design:**

Seventy‐three consultations between 16 clinicians and 47 patients were video‐recorded, transcribed and analysed using conversation analysis.

**Results:**

In most cases, patients do considerable interactional work to persuade clinicians of the validity of their concerns regarding breast asymmetry, and clinicians legitimize these concerns, aligning with patients. In a significant minority of cases, patients appear more accepting of their treatment outcome, but clinicians prioritize symmetry or treat symmetry with the presence of breast tissue as normative, generating misalignment between clinician and patient.

**Conclusion:**

Current clinical communication guidelines and practices may inadvertently reinforce culturally normative assumptions regarding the desirability of full, symmetrical breasts that are not held by all women. Clinicians and medical educators may benefit from detailed engagement with recordings of clinical communication like those analysed here, to reflect on which communicative practices may work best to attend to a patient's individual stance on breast symmetry, and optimize doctor‐patient alignment.

## 1 INTRODUCTION

Breast cancer is the most common occurring malignancy in women worldwide, accounting for 25.4% of all female cancers diagnosed in 2018.^1^ Surgery is the main treatment, with 81% of women undergoing mastectomy (total removal of breast) or lumpectomy (partial removal) procedures.^2^ UK guidelines state that all women should be offered cancer surgery provided this is not precluded by a significant comorbidity.^3^ The type of surgery elected depends on the grade, stage and location of the tumour, the size of the patient's breasts and their individual preference.^4^

Treatment and recovery for breast cancer are complex and multifaceted.^5^, ^6^ Insomuch as the disease presents a direct threat of mortality, it is also an assault on a particular body part deemed central to femininity and normative ideals relating to feminine beauty standards.^7^, ^8^ Surgical interventions typically involve lumpectomy with breast‐conserving surgery or mastectomy with or without reconstruction^9^ and may result in loss of one or both breasts, scarring, disfigurement and breast asymmetry.^10^, ^11^ The journey through diagnosis, treatment and recovery and into survivorship can present significant challenges to a patient's psychosexual health; her identity, body image, sexuality and confidence become subject to flux, uncertainty and change.^12^, ^13^

The contemporary goals of breast cancer surgery are no longer limited to cure alone, as the quality of cosmetic outcome is now considered a key clinical priority.^14^, ^15^ Given the centrality of breasts to many women's identity, body image, sexuality and self‐esteem, attempts to restore a patient's pre‐operative breast aesthetic and achieve breast symmetry are widely recognized as integral to surgical procedures.^16^, ^17^, ^18^ However, evidence suggests that following primary surgery for cancer, many women experience post‐operative difficulties relating to their breasts' overall appearance, including asymmetry.^19^, ^20^ A UK national audit of mastectomy and reconstruction reports that just 59% of patients were satisfied with their post‐operative appearance when looking in the mirror naked.^21^ In other studies, 77% of women cited body image and attractiveness,^22^ and 88.2% reported appearance^23^ as major treatment‐related concerns: all of which may negatively impact upon a patient's quality of life, with and beyond the disease.

Reconstructive surgery is thought to help women readjust to life and restore a sense of ‘normality’ after a breast cancer diagnosis.^24^, ^25^ Such procedures are referred to as a process rather than a singular event, often requiring more than one operation.^26^ Surgery to correct post‐operative appearance irregularities is common: one in five women who had primary breast‐conserving surgery in England were reported to need reconstructive surgery within three months of their initial operation,^27^ and approximately half of patients will require additional surgery within ten years after implant‐based reconstructions.^14^

Clinical guidelines advise that surgical procedures to optimize breast symmetry are available without time restrictions.^26^ However, there are reports of regional variations and unequal access to reconstructive services across the National Health Service (NHS) in England,^28^, ^29^ with limits placed on the number of surgeries a patient can have to complete breast reconstruction, the availability of balancing or symmetrization surgery to the unaffected breast and the time in which these procedures must be completed.^17^, ^26^

Quality of cosmetic outcome is viewed clinically as a critical endpoint of breast cancer surgery,^3^, ^15^ yet feminist literature problematizes this focus, arguing that breast cancer is viewed as an illness journey through which women are expected to reclaim not only their health, but also their pre‐cancer identity and appearance.^30^, ^31^ Consequently, aesthetic standards governing reconstructive procedures can be unrealistic, constructing healthy bodies as aligning with normative, idealistic concepts of ‘traditional femininity’^32^, ^33^—for example, the beauty ideal to appear full‐breasted and symmetrical. Feminist literature suggests that women whose breasts appear asymmetrical, or who lack one or both breasts, are culturally positioned as lying outside of feminine beauty standards.^34^, ^35^ For these reasons, research suggests that breast reconstruction may not be a ‘universal panacea’ for the emotional and physical consequences of breast cancer,^36^ but rather a complex, and at times paradoxical, psychological process for many women in breast cancer care.^37^

The ways patients and clinicians talk about the impact of breast cancer surgery on a patient's quality of life can profoundly influence their ability to live well, with and beyond the disease.^38^, ^39^ Effective communication is widely acknowledged as central to high‐quality cancer care and promotes patient satisfaction, psychological functioning and health‐related quality of life.^40^, ^41^, ^42^ However, patients cite communication as the one aspect of cancer care most in need of improvement^43^: they report that clinicians often gloss over the adverse psychological impact of surgery‐related changes to the appearance of their breasts^44^, ^45^ and that they encounter personal barriers to raising issues surrounding sexuality, body image and appearance due to embarrassment, or a belief that such matters are irrelevant in the wider context of cancer and mortality.^46^, ^47^

Previous research on communication about post‐operative breast appearance has primarily used satisfaction questionnaires and post hoc interviews with patients and clinicians.^38^, ^48^ These methods rely on participant memories of interaction that do not always accurately represent what happened, neglecting specific, contextualized details of real‐life clinical interactions, which are often ‘messier’ and more nuanced than retrospective accounts of experience.^49^ Despite the prevalence of post‐operative breast asymmetry amongst patients and the varied stances on the use of reconstructive surgery to help ‘restore’ a patient's pre‐operative breast aesthetic and achieve better symmetry, we do not know if and how these discussions play out in practice.

In this paper, we aim to address this evidence gap by identifying what can be learned from the fine‐grained analysis of video recordings of real clinical communication about breast symmetry in a breast cancer clinic. We will develop an understanding of how this topic unfolds in discussion by examining the interactional practices used by patients and clinicians to manage talk: focusing on not just what is said, but how it is said.

## 2 DESIGN

The data for this study were collected as part of a larger project that examines clinical communication about the psychosexual consequences of breast cancer and its treatment in an NHS breast cancer unit in the north‐west of England.

### 2.1 Recruitment and participants

Ethical approval was granted by the NHS's Research Ethics Committee. Forty‐seven patients (between 29 and 83 years of age) and sixteen clinicians (three breast surgeons, five breast care nurses, one oncologist, four clinical nursing staff and three health‐care assistants) were recruited using convenience sampling.

The breast unit's administrative team sent study information and consent forms to clinic patients with forthcoming appointments. Clinicians were recruited on‐site by the first author, who consented patients on the day of their appointment. Patients agreed to the recording of one to three consultations and clinicians to at least one consultation. Recruitment rates were 100% for clinicians and approximately 65% for patients.

The clinician sample comprised any member of the breast unit's multidisciplinary team who were involved in direct communication with patients. Including clinicians from diverse specialities afforded a rich picture of the differing contexts in which communication occurred. Patient inclusion criteria were female patients over the age of 18 years with a primary diagnosis of breast cancer. Male and transgender patients were excluded, as both groups warrant a separate research focus given the potentially diverse nature of their illness experience.^50^, ^51^

### 2.2 Data collection

Between 2017 and 2018, 73 consultations (totalling 30+ hours of video footage) were recorded. These included pre‐ and post‐operative consultations, reconstruction clinics, oncology clinics and adjuvant treatment consultations. Complex clinics (where patients return to the breast unit after being discharged to discuss ongoing effects of surgery) were also captured. Out of the 73 recordings, 26 were repeat visits. Diagnostic and primary results clinics were not included due to the potentially upsetting nature of these appointments. A video camera was located in the consultation room in advance of each appointment by the first author, who was not present during filming.

### 2.3 Data analysis

Recordings were viewed repeatedly and transcribed verbatim by the first author, producing over 550 pages of transcript. The corpus was then systematically searched for instances that contained discussions relating to breast symmetry, including talk about the similarity or difference in the shape, position, size or density of one breast in relation to the other.^15^ These discussions were most prominent in consultations that took place after the patient's initial cancer surgery.

**Figure 1:**
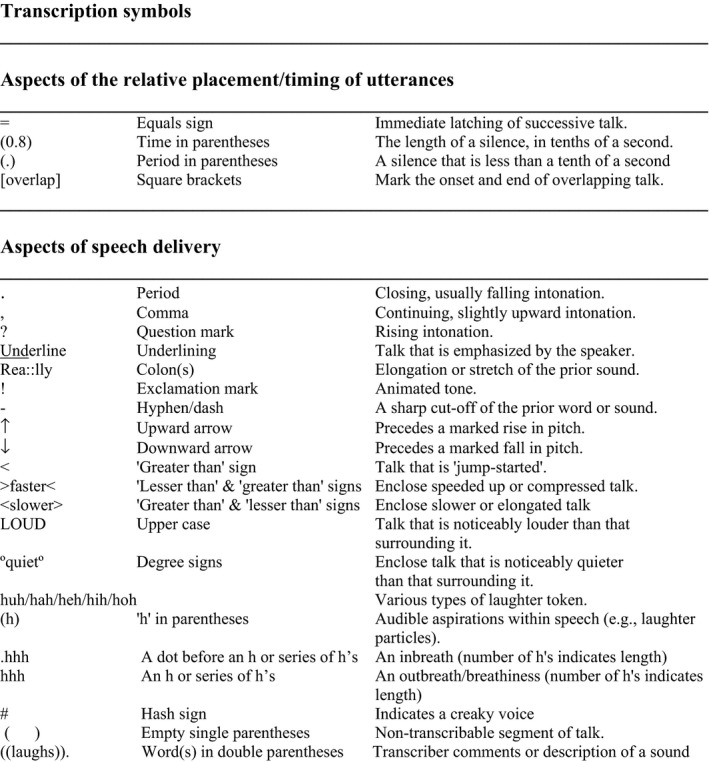
Transcription symbols—adapted from Jefferson^52^

Twenty‐seven relevant instances were identified from consultations between 22 different patients and seven clinicians. These instances were transcribed in detail using conversation analytic conventions (see Figure 1)^52^ and analysed using conversation analysis [CA].

Conversation analysis is grounded in the theoretical framework of ‘ethnomethodology’: the study of ‘members’ methods' for producing their everyday affairs,^53^ translating it into an empirical approach that examines the detailed and patterned organization of interactions in natural settings. CA has been used to great effect to identify communicative practices in clinical settings and to inform clinical communication,^54^, ^55^, ^56^ including the way delicate topics, such as weight, sex and cancer, are managed.^49^, ^57^

Analyses proceeded as follows: taking each instance in turn, transcripts were read alongside the original video in order to identify the main actions or ‘practices’ involved in talk about breast symmetry. Instances were analysed in greater detail by considering the non‐verbal actions, words, phrases and grammatical composition of those practices, and their relative position in the sequence.^49^, ^58^ The validity of CA findings is established by maintaining focus on data‐internal evidence and participants' orientations to one another's actions: each successive turn provides evidence for how the prior speaker's turn has been understood.^59^

## 3 FINDINGS

Analysis identified two main ways in which breast symmetry is discussed:

1. Patients do significant interactional work to persuade clinicians of the validity of their concerns about breast asymmetry, and clinicians legitimize, and attend to, these concerns (n = 19 instances from consultations between 16 different patients and seven clinicians. Fourteen of these patients were seeking further treatment).
2. Clinicians prioritize a symmetry agenda or treat it as normative, while patients are more accepting of asymmetry (n = eight instances, from consultations between six different patients and six clinicians. Two of these patients were seeking further treatment).

The six extracts below (featuring five different patients and seven clinicians) are representative of the two main communicative practices identified above. They exemplify common interactional features of these practices and important variations within each practice. Extract headers identify clinicians and patients by number.

### 3.1 Set One: Patients work to persuade clinicians of the validity of their concerns about breast asymmetry, and clinicians legitimize these concerns

The first set of instances represents the most common practice used by patients in discussions about breast symmetry across the data. In these sequences, patients do a significant amount of interactional work to persuade surgeons of the validity of their concerns about asymmetry and their need for reconstructive surgery, and surgeons attend to and legitimize these concerns.

The patient in Extract 1 (see Table 1) has had a lumpectomy of the left breast with breast‐conserving surgery. She is attending a reconstruction clinic three months after completing chemotherapy and radiotherapy treatment.

**Table 1:**
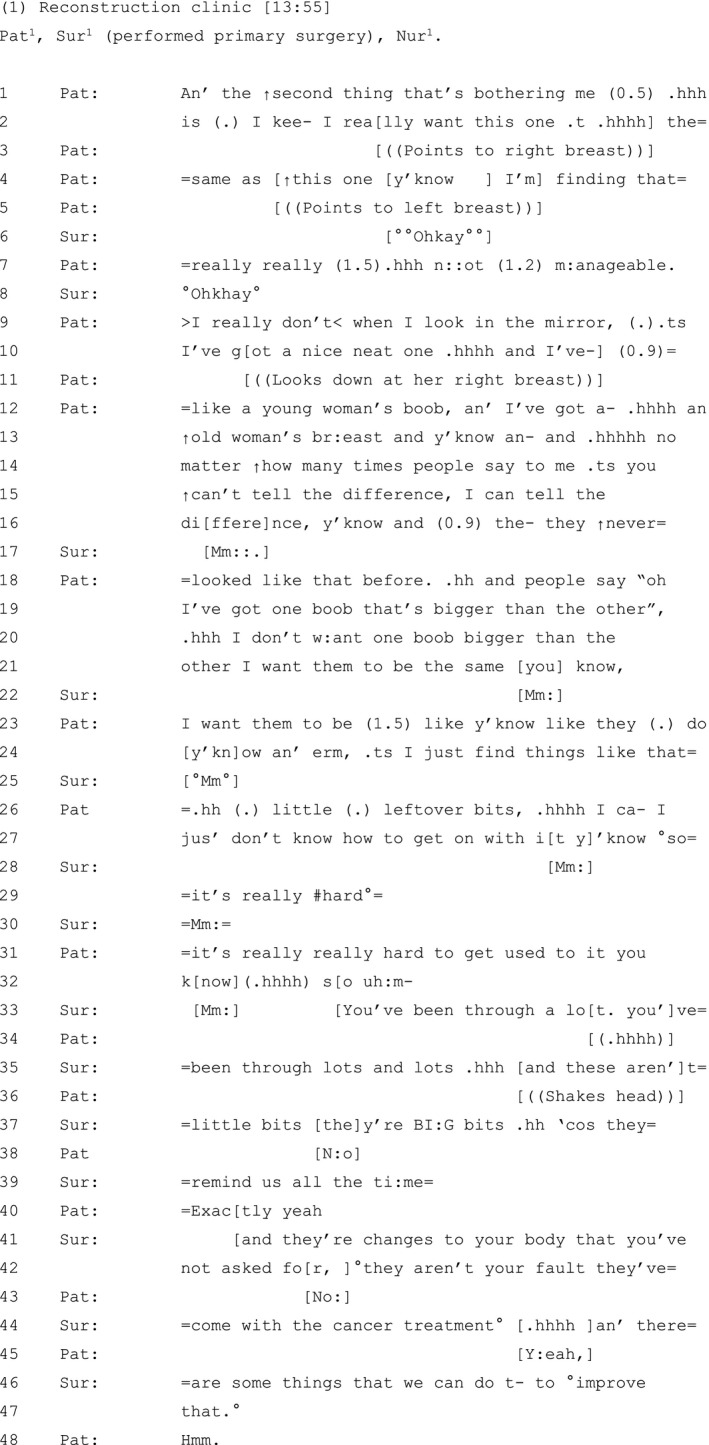
Extract 1

The patient's orientation to breast asymmetry, and wish for both breasts to be the same (lines 2‐4), is prefaced in negative terms, as the second of two concerns that are currently ‘bothering’ her (line 1).^60^ The patient expands her concern, describing a stark difference between her newly constructed ‘young woman's boob' and her healthy untouched ‘↑old woman’s br:east’ (lines 9‐13).

Using reported speech, the patient builds a contrast between what others say about her breast appearance and her own stance on the matter (lines 13‐21): others reportedly either cannot detect any difference between her breasts (lines 14‐15) or construct asymmetrical breasts as normative and everyday (lines 18‐19). The contrast allows the patient to index her epistemic authority and steadfastness vis‐à‐vis the views of others^61^: no matter what other people say to her, and how many times they say it, *she* can tell the difference (lines 15‐16) and desires both breasts ‘to be the same you know’ (line 21). She thereby invites the surgeon to witness how her subjective experience has been devalued, how this impacts her, and invites her support and alignment.

This reported dissatisfaction with her post‐operative appearance and assessment of its negative impact on her experience: it is ‘n::ot (1.2) m:anageable’ (line 7), could be heard as a potential complaint directed at the co‐present surgeon, who performed her surgery.^62^ This may account, in part, for why, after multiple attempts to seek the participation of the surgeon which are not always successful (note the multiple ‘you know’s’ in lines 13‐32), the patient works to minimize the significance of her concerns, reformulating her dissatisfaction with her breasts as ‘little (.) leftover bits’ (line 26). Research shows that patients may downplay the seriousness of aforementioned concerns in a bid to secure the clinician's acknowledgement of their presenting problem.^63^, ^64^ Here, by minimizing her concerns, the patient further invites the surgeon's support and alignment—which she now gets.^65^

The surgeon validates and demonstrates her ‘empathic attunement’ to the patient's concerns,^66^ by acknowledging the magnitude of her experience (lines 33‐35) and by explicitly challenging her minimizing assessment: ‘these aren’t little=bits, they’re BI:G bits’ (lines 35‐37).^66^ She attributes the patient's post‐operative difficulties to the cancer treatment (lines 42‐44) and proceeds to explore surgical options to address this (lines 44‐47).

Extract 2a (see Table 2) shares some similar features. This patient has had a unilateral lumpectomy of her left breast with breast‐conserving surgery. She is attending a complex clinic twelve months after being discharged from the breast unit.

**Table 2:**
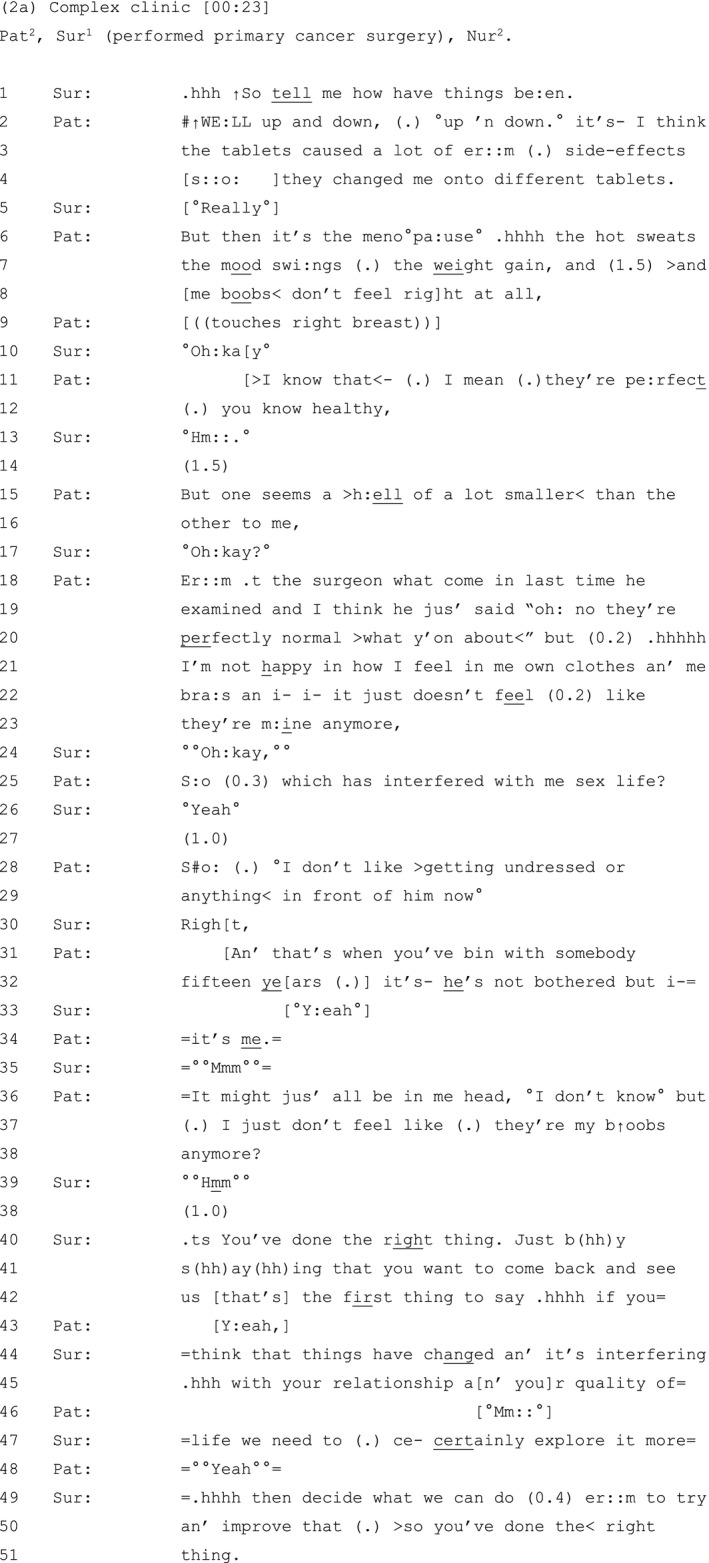
Extract 2a

Similar to Extract 1, the patient reports her current unhappiness with her post‐operative breasts: ‘me boobs< don’t feel right at all’ (line 8), at the end of a list of other concerns about treatment‐related side effects (lines 6‐7).^60^ Although she concedes that her breasts are perfectly healthy in terms of being cancer free (lines 11‐12), she attends to their far‐from‐perfect asymmetrical appearance: ‘But one seems a >h:ell of a lot smaller< than the other to me’ (lines 15‐16).

Like the patient in Extract 1, this patient expands her concerns by reporting the contrasting views of others. The previous surgeon's assessment: ‘they’re perfectly normal >what y’on about<’ (lines 19‐20), is built as dismissing her concerns about asymmetry and invites the current surgeon to witness how her subjective experience has been devalued. She also reports the view of her partner as ‘not bothered’ by the appearance of her breasts (line 32). Once more, the patient uses these contrasting views as a platform to bolster her own stance on asymmetry: regardless of what others think, it is negatively impacting her body image, sex life and confidence in getting undressed in front of her partner (lines 21‐29).

Here again, the patient minimizes the significance of the concerns she has just described, this time using the self‐deprecation: ‘It might jus’ all be in me head’ (line 36). We know that self‐deprecations can be used by speakers to 'do' identity work, by presenting themselves as reflexive, analytic beings, able to recognize the potential criticism of others (in this case, that the patient may be ‘imagining it’), at the same time as inoculating themselves against, and preventing, just such criticism.^65^ It appears to be used here to invite clinician alignment at precisely the point where such alignment may be lacking (note the barely audible ‘°°Mmm°°’ on line 35).^67^, ^68^ As before, the surgeon now validates the patient by reassuring her that she has done ‘the right thing’ (lines 40‐47), before attending to the treatment options that can ‘improve that’ (line 50).

To summarize, extracts 1 and 2a unfold in a similar way. The patient:

1. Builds her post‐operative breast asymmetry as a concern (eg ‘the ↑second thing that’s bothering me’ [Extract 1, line 1], and ‘me boobs don’t feel right at all’ [Extract 2a, line 8]);
2. Works to persuade the surgeon of the validity of her subjective experience, by building a contrast between her own views (eg ‘I want them to be the same’ [Extract 1, line 21], and ‘one seems a >h:ell of a lot smaller< than the other to me’ [Extract 2a, lines 15‐16]), and the views of others (eg ‘people say “oh I’ve got one boob that’s bigger than the other” [Extract 1, lines 18‐19], and ‘he jus’ said “oh: no they’re perfectly normal >what y’on about<”’ [Extract 2a, lines 19‐20]);
3. Minimizes the significance of the concern she has just built (‘little (.) leftover bits’ [Extract 1, line 26], and ‘It might jus’ all be in me head’ [Extract 2a, line 36]);

The surgeon responds by:

- Validating the patient's concerns about asymmetry, challenging their minimizing or self‐deprecatory assessments (eg ‘these aren’t little==bits they’re BI:G bits’ [Extract 1, lines 35‐37], and ‘You’ve done the right thing’ [Extract 2a, line 40]), and;
- Discussing additional surgical options to address the patient's concerns.

Later in the same consultation (Extract 2b), the patient expands on her aforementioned concerns about asymmetry, and we see a variation on the above sequence (see Table 3):

**Table 3:**
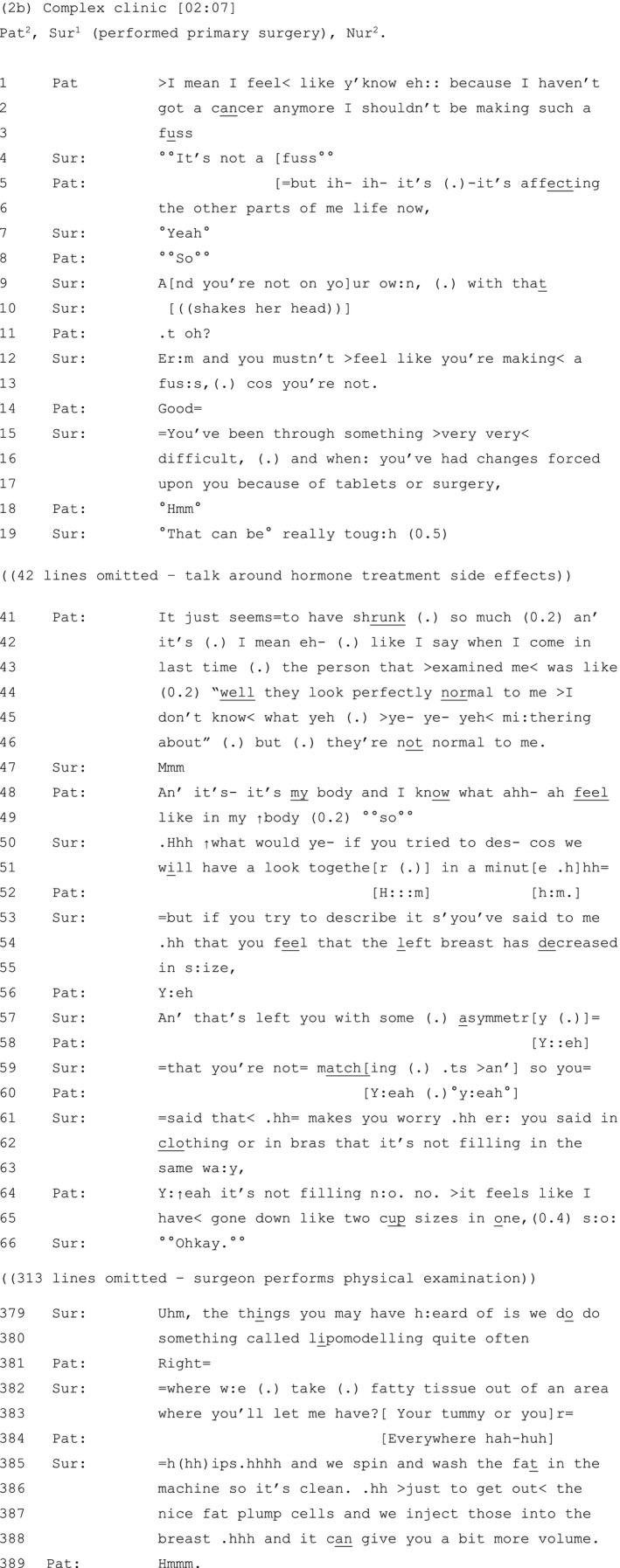
Extract 2b

Once again, the patient:

1. Minimizes the significance of the concerns she has expressed, in this case through the self‐deprecatory meta‐comment,^65^ ‘shouldn’t be making such a fuss’ (lines 2‐3);

The surgeon:

- Validates the patient's concerns by challenging her claim to be making ‘a fuss’, for example ‘°°It’s not a fuss°°’ (line 4), and ‘you mustn’t >feel like you're making< a fus:s’ (lines 12‐13), and by attributing the difficulties she experiences to the cancer treatment (lines 15‐17);

The patient:

- Works to persuade the surgeon of the validity of her subjective experience by building a contrast between the invalidating views of another clinician: ‘the person that >examined me< was like (0.2) "well they look perfectly normal to me >I don’t know< what yeh (.) >ye‐ ye‐ yeh< mi:thering about" (lines 44‐46) and her own views: ‘it’s my body and I know what ahh‐ ah feel like in my ↑body’ [lines 48‐49]).

Finally, the surgeon:

- Further validates the patient's concerns by summarizing and seeking confirmation of her understanding of those concerns (lines 53‐63). Following a physical examination of the patient, she proceeds to discuss reconstructive options (lines 379‐388).

Extract 3 (see Table 4) demonstrates the robustness of the sequence described so far. This patient is attending a delayed reconstruction clinic some twelve months after completing adjuvant treatment. She had a unilateral mastectomy of her left breast, opted not to have immediate reconstruction and currently wears a breast prosthesis.

**Table 4:**
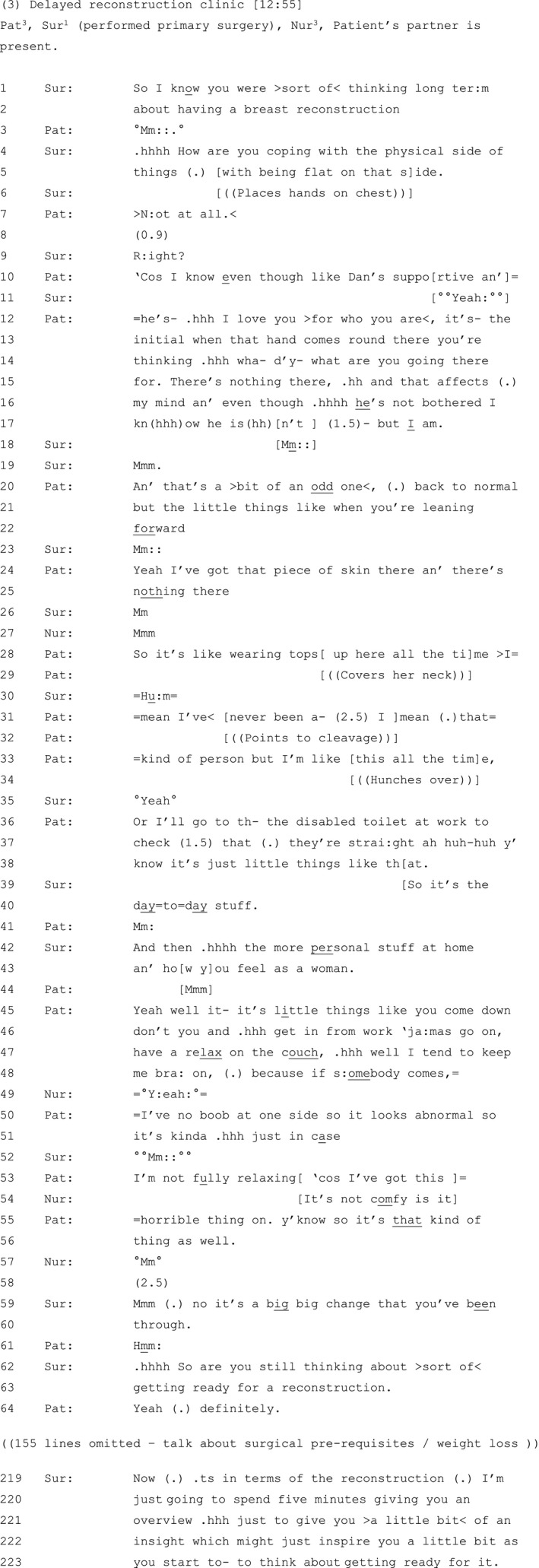
Extract 3

Here, the patient's desire for a breast reconstruction is made relevant by the surgeon's reference to a prior discussion (lines 1‐2). The surgeon makes asymmetry explicit by asking the patient how she is currently ‘coping’ with being flat on one side (lines 4‐6). The use of the word ‘coping’ suggests that the surgeon is treating breast asymmetry as something negative that requires management.

The patient:

1. Builds her postoperative breast asymmetry as a concern by responding that she is not coping ‘at all’ (line 7);
2. Accounts for her response, and works to persuade the surgeon of the validity of her subjective experience, by building a contrast between the views of her partner, who reportedly tells her ‘I love you >for who you are<’ (line 12) and is not ‘bothered’ (line 16) by the absence of her left breast, and the steadfastness of her own view: ‘but I am’ (line 17);
3. Minimizes the significance of the concerns she has just expressed, by reformulating her situation as ‘a >bit of an odd one<, (.) back to normal but the little things…’ (lines 20‐21). She unpacks what she means by her minimizing assessment ‐ ‘little things’ (lines 21‐37), repeating it on a further two occasions (lines 38 and 45) as she further unpacks her concern: ‘I’ve no boob at one side so it looks abnormal’ (line 50).

The surgeon responds by:

- Validating the patient's concerns about asymmetry, reformulating ‘little things’ as ‘day=to=day stuff’ (line 40), ‘personal stuff’ (line 42), and ‘how you feel as a woman’ (line 43), and de‐trivializes the patient's experience by reminding her of the ‘big big change’ (line 59) that she has been through.
- Treating the patient's concerns as understandable, justified and deserving of a solution, by enquiring whether the patient is still considering reconstructive surgery (lines 62‐64), and later outlining the reconstructive options available (lines 219‐223).

So far, we have shown how patients engage in considerable interactional work to persuade clinicians of the validity of their concerns regarding asymmetry. As part of this work, patients refer to other, contrasting viewpoints on their asymmetry, thereby demonstrating the strength of their subjective experience in the face of such viewpoints. In each case, they downplay the seriousness of their aforementioned concerns in a bid to secure the clinician's acknowledgement of their presenting problem,^63^, ^64^ further inviting support and alignment. In each case, the clinician empathizes with and validates the patient's concerns, while progressing those concerns as grounds for a potential solution: reconstructive surgery.

The remaining instances represent a significant minority of cases. Here, we see evidence for misalignment between clinician and patient, as clinicians prioritize a symmetry agenda, or treat symmetry as normative, while patients are more accepting of asymmetry.

### 3.2 Set Two: Patients appear more accepting of their treatment outcome, but clinicians prioritize symmetry or treat symmetry with the presence of breast tissue as normative

Extract 4 (see Table 5) is from a follow‐up consultation with a patient who has had surgery to replace a temporary implant with a permanent one.

**Table 5:**
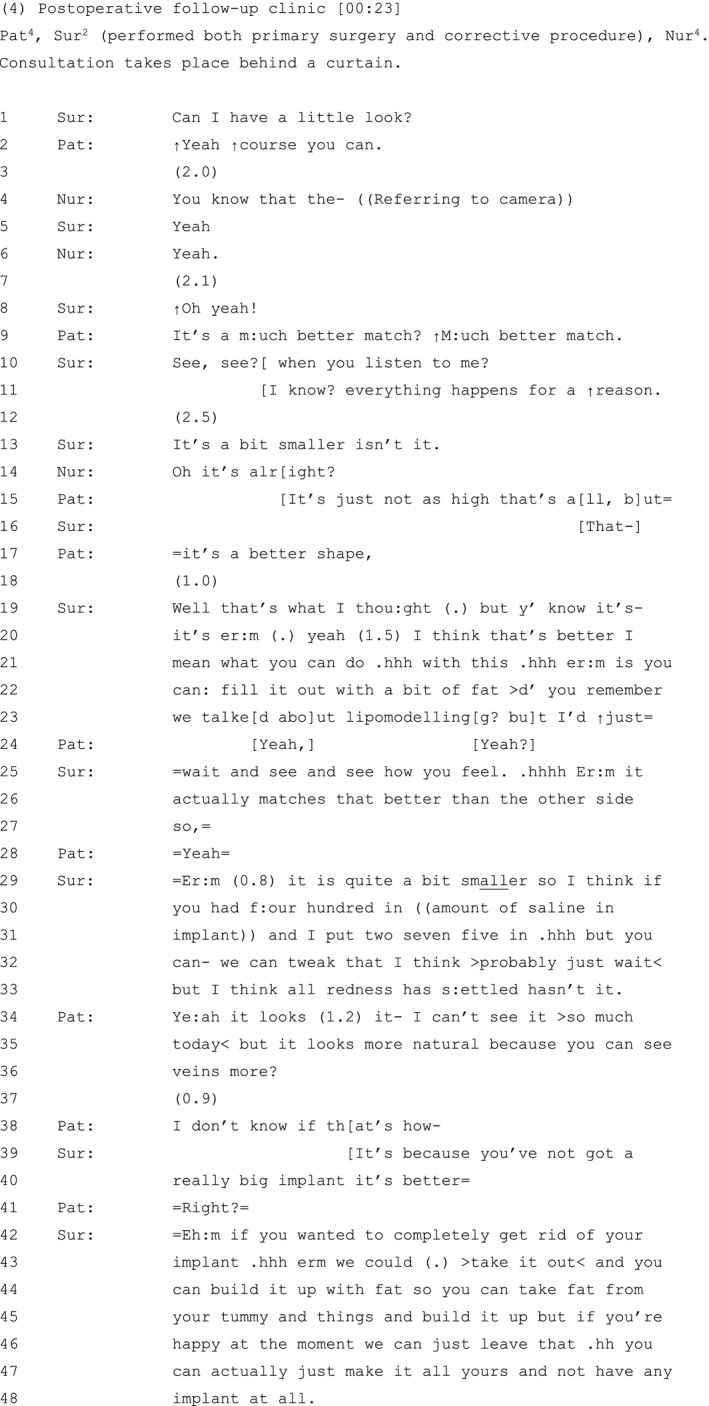
Extract 4

The sequence opens with an examination of the patient's breasts. The surgeon's initial response to the patient's post‐operative appearance ‘↑Oh yeah!’ (line 8) conveys approval of the overall aesthetic of the patient's breasts. The patient initially aligns with this position, orienting to the improved symmetry in her repeated assessment that it is a ‘m:uch better match?’ (line 9). The surgeon indicates that this positive outcome is a result of the patient adhering to her advice on surgical options (line 10), which the patient endorses (line 11).

At this point then, both parties appear aligned in their approval of the more symmetrical treatment outcome. However, following a significant gap, during which the surgeon continues to examine the patient (line 12), she identifies problems with her work: ‘it’s a bit smaller isn’t it’ (line 13). Built as a negative interrogative, this assessment is grammatically tilted towards an agreeing response (eg ‘yes, it is’).^69^ However, both nurse and patient resist endorsing the surgeon's negative assessment, instead aligning in responses that serve to minimize concerns about asymmetry (lines 14‐17), and reassure the surgeon of their approval of the outcome. The nurse's ‘oh‐prefaced’ assessment that the breast is ‘alright’ (line 14) rebuts the surgeon's negative assessment.^70^ Likewise, the patient acknowledges that although her reconstructed breast is not in the same position as her healthy breast, it is, nonetheless, a ‘better shape’ (line 17).

Interestingly, instead of aligning with the nurse and patient (by agreeing that they are right, for example), the surgeon asserts her epistemic primacy over the assessment of the clinical outcome by building their position as one that was consistent with an opinion she had already formed, independently of, and prior to, the nurse and patient stating their views; ‘well that’s what I thou:ght’ (line 19).

Despite evidence that both nurse and patient appear more positive about the treatment outcome, and status quo, the surgeon nonetheless maintains and progresses her symmetry agenda by describing ways in which the asymmetry may be surgically addressed (lines 21‐48).

We note similar misalignment between a different surgeon and patient in Extract 5 (see Table 6). This patient is attending a follow‐up clinic after a unilateral mastectomy with immediate reconstruction using an expander implant.

**Table 6:**
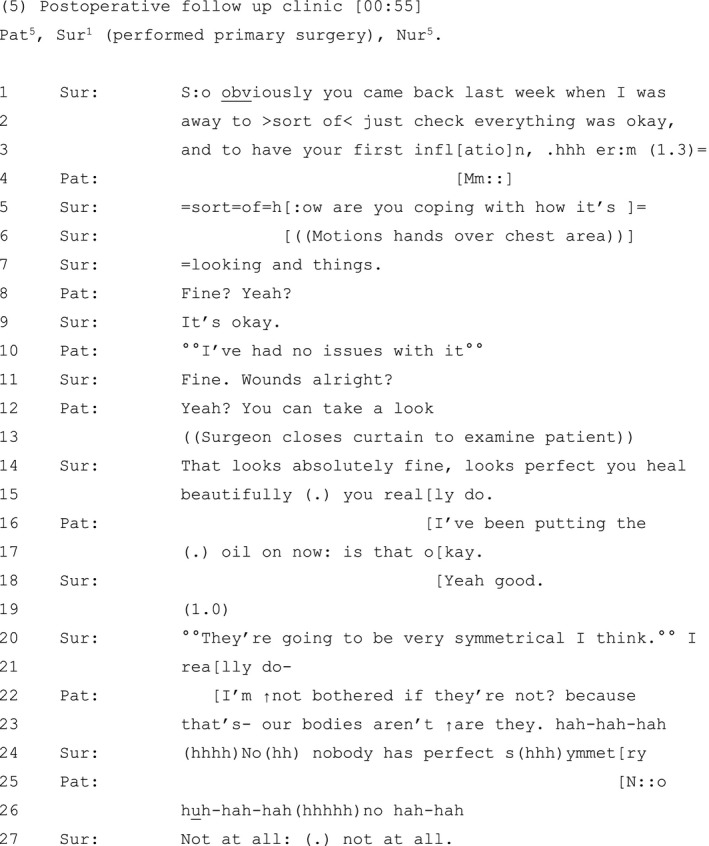
Extract 5

The surgeon asks the patient how she is ‘coping’ with her post‐operative breast appearance (line 5), and she responds that she is ‘fine’ (line 8) and has ‘had no issues with it’ (line 10). Clinician and patient are aligned over the patient's positive outcome in terms of wound healing (lines 11‐18).

However, the surgeon's later announcement that the patient's breasts are going to be ‘very symmetrical’ (line 20) is built for an approving response in which the assessment is treated as good news (e.g. ‘oh, that’s great!’).^71^ However, the patient's response does not align. Instead, she actively challenges the fundamental basis of the surgeon's assessment, on the grounds that achieving very symmetrical breasts is not her concern, *and* does not reflect the reality of our bodies, where symmetry is non‐normative: ‘I’m ↑not bothered if they’re not? because that’s‐ our bodies aren’t ↑are they’ (lines 22‐23). The patient's response may be heard as resistance to medical authority.^63^, ^72^ Indeed, her challenge is a delicate one to bring off interactionally, and something she manages by grammatically composing her turn for agreement: ‘our bodies aren’t [symmetrical] are they’, (line 23), which it gets ‐ ‘No(hh)’ (line 24), and through post‐completion laughter particles (line 23, see also lines 25‐26), which are often used by patients to deal with delicate aspects of medical interaction, including challenges to authority.^73^

Standing corrected, the surgeon aligns with the patient, diffusing delicacy by agreeing with her position, joining with her laughter^74^ and explicitly referencing the non‐normativity of symmetrical breasts ‘(hhh)No(hh) nobody has perfect s(hhh)ymmetry’ (line 24). Both, in solidarity, reconfirm the correctness of the patient's position (lines 25‐27).

The final extract (see Table 7), from a nurse‐led adjuvant treatment follow‐up consultation, is an interesting case. This patient had a bilateral mastectomy, has opted not to have reconstruction and does not wear any breast prostheses.

**Table 7:**
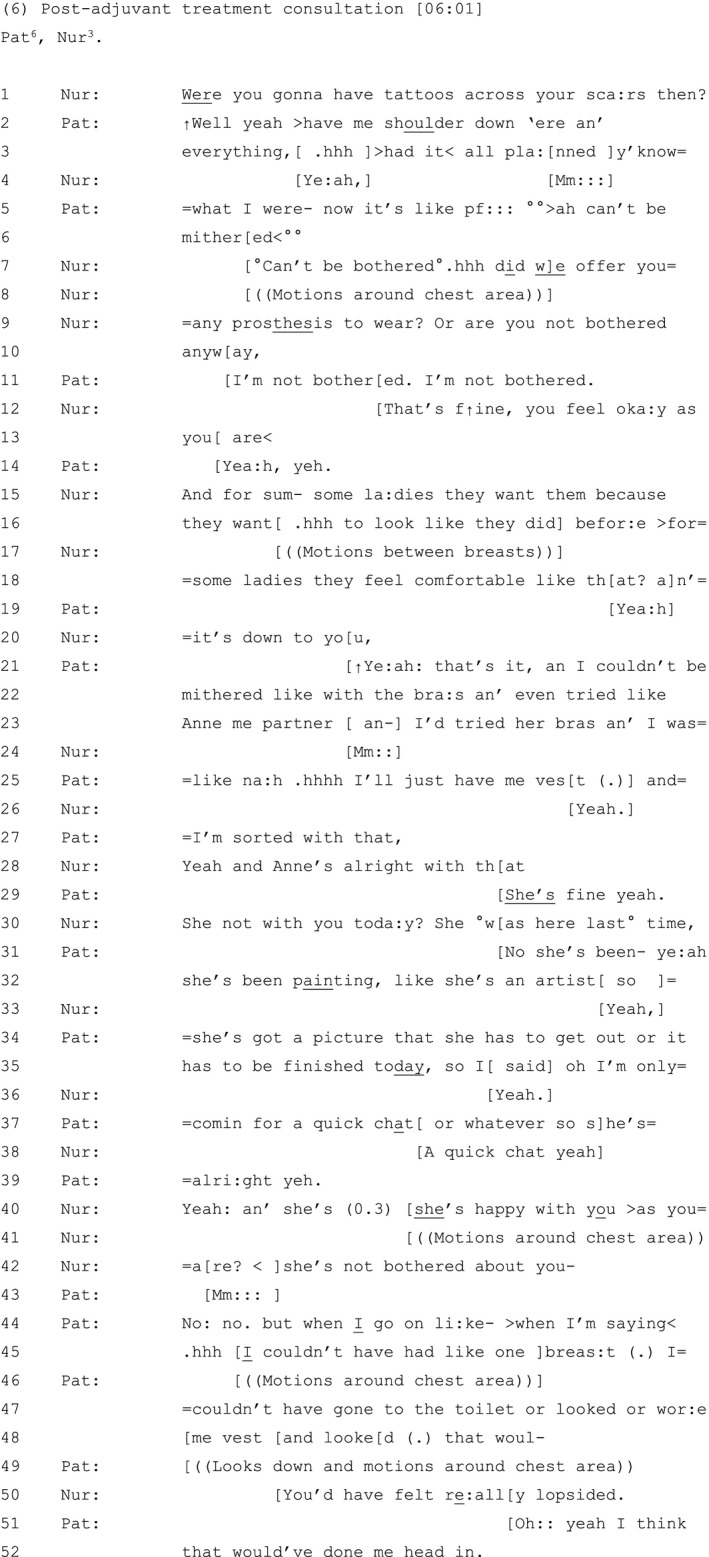
Extract 6

This patient treats symmetry without the presence of breast tissue or a prosthesis, as an acceptable outcome: she repeatedly makes clear that she is ‘not bothered’ about wearing a prosthesis (line 11). By contrast, the nurse treats voluntary flat‐chestedness as an accountable, and non‐normative matter, by gently checking and re‐checking whether the patient and her partner are satisfied with her post‐operative appearance (lines 12‐13, 28 & 40‐42).^57^

She contrasts the patient's own preference not to wear a prosthesis with ‘some la:dies’ (line 15) who want one because ‘they want.hhh to look like they did befor:e’ (lines 15‐16), comparing this to other ladies who ‘feel comfortable like that?’ (line 18). The nurse reinforces the patient's agency in her decision making: ‘it’s down to you’ (line 20).

The patient agrees with the nurse (line 21), validating her decision as based on personal experience (lines 21‐22). She refers to her partner, Anne, who had a diagnosis of breast cancer the year prior and now wears a prosthesis: she has ‘even tried’ Anne's prosthesis but thought ‘na:h:’ (lines 22‐25). Similar to patients in Set One, this patient refers to the position of an ‘other’ as a means to bolster her own stance on her appearance preference: while her partner uses a bra and breast prosthesis, this patient is happy with the current status quo: ‘I’ll just have me ves:t (.) and I’m sorted with that’ (lines 25‐27).

Although the patient has arguably made her position, and her partner's acceptance of that position, clear (line 29), the nurse notes and seeks an account for Anne's absence from the appointment (line 30). The patient provides this account (lines 31‐37), and the nurse checks again whether Anne is okay with the patient's appearance ‐ ‘she’s happy with you >as you are?’ (lines 40‐42). Interestingly, flat‐chestedness is not explicitly mentioned by either party; instead, the nurse alludes to it by motioning around the breast area to indicate that it is the patient's lack of breasts that is the target of her enquiry (line 41). The patient confirms that her partner is happy with her as she is (line 43) and further unpacks her decision to remain flat‐chested by spelling out the state of affairs she would have found unacceptable: ‘I couldn’t have had like one breas:t (.) I=couldn’t have gone to the toilet or looked or wor:e me vest and looked’ (lines 45‐48). The nurse demonstrates her attunement with the patient by collaboratively completing her turn^75^: ‘you’d have felt re:ally lopsided’ (line 50). For this patient, no breasts are better than asymmetrical breasts.

## 4 DISCUSSION AND CONCLUSION

### 4.1 Discussion

Our findings show that in the majority of instances of clinical communication about breast symmetry (n = 19 out of 27), patients engage in considerable interactional work to persuade clinicians of the validity of their concerns about, and subjective experiences of, post‐operative asymmetry, and clinicians legitimize and attend to these concerns. However, in a significant minority of cases (n=8 out of 27) there is misalignment between patient and clinician, as patients appear more accepting of their treatment outcome, but clinicians nonetheless prioritize symmetry or treat symmetry with the presence of breast tissue as normative.

Findings have implications for clinical training and practice:

In Set One, there is clear evidence that clinicians skilfully validate women's experiences and concerns about asymmetry, challenge their attempts to minimize those concerns and align with them by treating those concerns as reasonable, ‘doctorable’ and requiring additional surgery.^69^ However, it is striking just how hard patients work, in interactional terms, to achieve this. Indeed, the robustness of the action sequence identified suggests that patients routinely treat this interactional effort as necessary.^76^ They are at pains to demonstrate a reflexive orientation to the contrasting views of others, which serves to reinforce the strength of their experience and need for intervention in the face of alternative viewpoints. However, they also minimize or downplay the seriousness of their aforementioned concerns (eg ‘little leftover bits’ [Extract 1], ‘all in me head’ [Extract 2a], ‘making such a fuss’ [Extract 2b], ‘little things’ [Extract 3]). In this way, they orient towards, pre‐empt and deflect potential criticism for ‘complaining about trivialities’ in the context of surviving cancer, and secure the clinician's acknowledgement of their presenting problem.^63^, ^64^

This interactional work may be outcome‐relevant: research shows that patients can struggle to articulate their desires for particular interventions and may use various interactional methods to apply ‘subtle’, yet ‘persistent’ pressure for a certain treatment, without explicitly requesting it.^76^ Moreover, since one of the aims of these consultations is to decide whether surgical revision should be done, and surgeons are uniquely qualified to provide this intervention, the surgeon needs to be convinced that there is a problem. In fee‐for‐service health‐care systems, this may not be difficult to achieve, since there are potential financial gains to be made. However, in the UK, taxpayer‐funded, free‐at‐the‐point‐of‐service health‐care system, patients may fear they will not qualify for additional surgery, amidst reports of unequal access to reconstructive services across England.^28^, ^29^

Although these interactions fundamentally reflect the health‐care system in which they are situated, it is not inevitable that the burden of interactional effort in this context must always fall to patients. Findings indicate that it may prove beneficial for clinicians to pre‐empt, invite and assuage patients' concerns about asymmetry, and their need for validation, at the earliest opportunity in the consultation. For example, they could do this by acknowledging that it is both common and entirely acceptable for women to be dissatisfied with their post‐operative appearance and that this can be rectified surgically, if required.

The instances in Set Two, while fewer in number, underscore the importance of acknowledging patient diversity in breast cancer care. These cases demonstrate the communicative consequences of adopting the culturally normative assumption that achieving breast symmetry, and symmetry with the presence of breast tissue, may be the most desirable treatment outcome.

Here, it is clinicians who pursue an agenda that focuses on achieving better symmetry, or treat symmetry with the presence of breast tissue as normative. This agenda is in line with clinical guidelines that emphasize the cosmetic quality of surgery,^15^, ^77^ and the importance of ensuring patient satisfaction with post‐operative outcomes^3^, ^14^: There is a positive correlation between optimal post‐operative cosmesis, breast symmetry, high‐quality survivorship and overall psychological adjustment.^15^, ^78^ In this respect, our data reflect a certain pressure placed on clinicians to maximize patients' quality of life with and beyond the disease. However, this symmetry agenda also strongly reflects normative ideas relating to feminine appearance, which subscribe to the presence of full, ample and symmetrical breasts.^33^ By prioritizing symmetry, clinicians may inadvertently misrepresent the patient's individual stance on their post‐operative appearance, leading to the misalignment we see in Set Two, and potentially alienating some women.

Extracts in Set Two point towards the potential inadequacies of adopting a uniform approach to communication with patients about post‐operative appearance, and challenge notions that breast cancer is a cosmetic crisis for *all* women.^74^ Findings contribute to research that questions normative appearance conventions in breast cancer care,^34^, ^35^ as here, patients resist conventional pressures placed on women to restore their body's ‘normal’ appearance after surgery. Patients counter clinician concerns relating to asymmetry by outlining their satisfaction with their post‐operative appearance (Extract 4), questioning the achievability of perfectly symmetrical breasts (Extract 5) and implicitly resisting the idea that they need breast tissue to achieve an acceptable kind of symmetry (Extract 6). In so doing, these patients challenge discourses that pathologize bodies that fall outside of these parameters as physically deformed and incomplete.^34^, ^35^

Findings suggest that clinicians and medical educators may benefit from remaining alert to the possibility that patients possess stances towards breast symmetry that differ from normative constructs of ideal feminine appearance, and to actively work to treat those perspectives as valid.

### 4.2 Limitations

Data were collected from a single site, which restricts the claims that can be made about the generality of the interactional practices identified. The specific nature of this topic meant that breast surgeons predominate in our analysis. Future research may benefit from considering how expectations relating to post‐operative appearance are managed within pre‐operative appointments. Finally, our patient sample is predominantly white, heterosexual and post‐menopausal: a more diverse demographic may produce different results.

### 4.3 Conclusion

This study offers a snapshot of current practice in a breast cancer clinic. Post‐operative breast asymmetry is a common outcome for many women with breast cancer.^11^ However, current clinical communication guidelines and practices reflect the health‐care context in which they are situated and may inadvertently reproduce culturally normative assumptions regarding the desirability of full, symmetrical breasts that are not held by *all* women. Data point to the potential benefits of a fine‐grained consideration in clinical training and practice of individual patient interpretations of post‐operative appearance, and of being attuned to the possibility that for some women, asymmetrical breasts, or even no breasts, can be an acceptable surgical outcome.

Clinicians and medical educators may therefore benefit from detailed engagement with recordings of clinical communication like those analysed here, to reflect on which communicative practices work best to attend to a patient's individual stance on post‐operative appearance and breast symmetry, and optimize doctor‐patient alignment.

## CONFLICT OF INTEREST

There are no conflicts of interest to disclose.

## AUTHOR CONTRIBUTIONS

The design of the study was conceived by Speer in conjunction with Mace and Collins. Mace conducted the study for her PhD thesis under the supervision of Speer and Collins, secured access and ethical approval and recruited participants. Mace collected and transcribed all data, which were analysed by Mace in conjunction with Speer and Collins. Mace prepared the first version of the manuscript. All authors commented on and contributed to successive drafts.

## Data Availability

Raw research data are not shared, given the sensitivity of patient data and the ethical requirements governing this study.
